# ‘Overhaul Medicare and perhaps train us better’: a qualitative study of primary care general practitioners’ perspectives on how to implement the low back pain clinical care standards

**DOI:** 10.1136/bmjph-2025-002564

**Published:** 2025-09-08

**Authors:** Nathalia Costa, Chung-Wei Christine Lin, Fiona Blyth, Carmen Huckel-Schneider, Rachelle Buchbinder, Danijela Gnjidic, Aili Langford, Denise O’Connor, Carl Schneider, Simon French

**Affiliations:** 1 The University of Queensland, The University of Queensland Clinical Trials Capability (ULTRA) Team, Brisbane, Queensland, Australia; 2 Leeder Centre for Health Policy, Economics and Data, School of Public Health, The University of Sydney, Sydney, New South Wales, Australia; 3 Sydney Local Health District, The University of Sydney Institute for Musculoskeletal Health, Sydney, New South Wales, Australia; 4 Sydney School of Public Health, The University of Sydney, Sydney, New South Wales, Australia; 5 School of Public Health and Preventive Medicine, Monash University, Melbourne, Victoria, Australia; 6 School of Pharmacy, The Faculty of Medicine and Health, The University of Sydney, Sydney, New South Wales, Australia; 7 Department of Chiropractic, Faculty of Medicine, Health and Human Sciences, Macquarie University, Sydney, New South Wales, Australia

**Keywords:** Public Health, Qualitative Research, standards

## Abstract

**Introduction:**

In September 2022, the Australian Commission on Safety and Quality in Healthcare released the Low Back Pain Clinical Care Standards (‘Care Standard’). We aimed to explore general practitioners’ (GPs’) perspectives on how the Care Standard can be implemented, with a view to identifying what strategies could be used to do so.

**Methods:**

This qualitative study is underpinned by a critical realist philosophy. We interviewed 16 GPs across Australia with experience working with patients who present with low back pain (LBP). Interview questions were based on the Theoretical Domains Framework, and interview data were analysed using reflexive thematic analysis.

**Results:**

We identified three themes: theme 1—‘I have my own guidelines and ways of doing things’—guidelines and standards are not relevant for experienced GPs’ captures the perceived unnecessariness of seeking advice from guidelines and standards for LBP; theme 2—I agree with the standards but the system, clinicians and patients hinder my ability to enact them—reports factors impacting the will and ability of clinicians to implement the standards in practice; theme 3—Change the system and train us better—discusses potential strategies to implement the Care Standard: changing funding and infrastructure (eg, improving GPs’ reimbursement for time spent, investing in publicly funded allied health services); offering training and education (eg, continuing professional development courses, improving communication training in undergraduate courses), developing stakeholder relationships (eg, primary health networks, Health Pathways), supporting clinicians through interactive assistance (eg, reminders, embedding assessment tools in existing systems), engaging/educating patients and using evaluative strategies such as auditing.

**Conclusions:**

Our results suggest that the multidimensional nature of the challenges GPs face, and the strategies they suggest, calls for a multimodal and integrated approach to implementing the Care Standards, including system changes.

**WHAT IS ALREADY KNOWN ON THIS TOPIC**

- Variation in care for low back pain is a major issue in Australia and globally.
- A clinical care standard has been developed to reduce variation in care for low back pain in Australia, but general practitioners (GPs’) views on how it should be implemented are unknown.

**WHAT THIS STUDY ADDS**

- Our study indicates that the perceived unnecessariness of consulting standards, health system-related factors and other clinicians and patient-related factors hinder GPs’ ability to practise in alignment with care standards.
- Changes in funding and infrastructure, (continued) education, collaboration across stakeholders, interactive support and engaging patients can assist the implementation of care standards in practice.

**HOW THIS STUDY MIGHT AFFECT RESEARCH, PRACTICE OR POLICY**

- A multimodal and integrated approach is needed to improve care provided to patients with low back pain, including policy and system changes.

## Introduction

 Low back pain (LBP) is the most common musculoskeletal symptom for which patients consult general practitioners (GPs) in Australia, and the third most common symptom overall.^1 2^ It places a major economic burden on the Australian healthcare system, with costs exceeding AUD$4.8 billion each year.^3^ Alongside the financial impact of LBP is the epidemic of low-value care; for example, despite recommendations about limiting imaging to specific rare indicators,^4^ the number of Medicare Benefits Schedule-funded services for lumbar spine CT remains high.^5^ Likewise, while pharmacological therapies including the use of opioids are generally discouraged,^6^ the prevalence of long-term opioid prescribing in people with LBP and other musculoskeletal conditions has increased from 5.5.% (95% CI 5.2% to 5.8%) in 2012 to 9.1% (95% CI 8.8% to 9.7%) in 2018.^7^

To reduce unwarranted variation in practice and improve care for LBP, the Australian Commission on Safety and Quality in Healthcare released the Low Back Pain Clinical Care Standard (henceforth called ‘Care Standard’) in September 2022.^8^ The Care Standard covers the care that should be provided to patients aged 16 years and over who present with a new acute episode of LBP. It was drafted over a series of meetings by representatives of the professions who manage LBP, consumer advocacy groups, NPS MedicineWise (a not-for-profit organisation that provides evidence-based and consumer-centred information), independent experts, Commission representatives, as well as being reviewed and endorsed by 19 professional associations.^8^

While the Care Standard is a crucial step towards improving care for LBP in Australia, it can be difficult to ensure successful implementation into practice.^9^ Implementation strategies informed by end-users have the potential to provide insights on how standards can be successfully adopted in practice.^10 11^ As GPs are a common first point of contact for people with LBP,^2^ we sought to understand their perspectives on how the Care Standard can be implemented in general practice while considering the complexities and nuances of real-world contexts.

## Methods

We conducted a descriptive qualitative study and gathered data through interviews. Our study was underpinned by a critical realist philosophy, which posits the world is an open system where events or effects result from complex and often unobservable (yet real) interactions between human agency and social structures.^12^ Such an approach enables an understanding of under what circumstances and how the Care Standard may be used in general practice, drawing from GPs’ experiences to theorise strategies that may support its implementation.^13 14^

### Research team

We are an interdisciplinary group of researchers with a special interest in LBP. With an applied focus, we approached this study by merging our expertise in public health, clinical epidemiology, policy, pharmacy, physiotherapy, chiropractic, general practice, rheumatology and implementation science. Additionally, six of us have experience working clinically with people with LBP, five of us have experienced LBP and two of us were involved in the development of the Care Standard.

### Participant recruitment and procedures

We recruited registered GPs in Australia with experience caring for people presenting with LBP through social media platforms and an Australian-wide recruitment service (TKW Research). Recruitment was both convenience-based and purposeful, as we aimed to include GPs with different levels of experience, from both metropolitan and regional areas. Those who provided consent to participate were interviewed by NC via video conference for 45–65 min (average: 55 min). NC is a senior qualitative researcher with extensive experience conducting interviews—she had conducted over 200 interviews with clinicians, people with lived experience and other key stakeholders before interviewing GPs for the present study. She has a PhD in physiotherapy and postdoctoral studies at the interface of clinical sciences and sociology and in health policy. During the interview, NC shared at least two of the eight quality statements from the Care Standard (see online supplemental figure 1) via the screen share function and asked questions that encouraged participants to discuss their views on them and how they could be implemented in Australian general practice. Different quality statements were randomly shared with different participants and each quality statement was discussed with at least three participants. Interview questions were developed by NC, SF and DO’C, reviewed by coauthors and drew on the Theoretical Domains Framework (TDF)^15^ (see interview guide in online supplemental file 2). Interviews were conducted approximately 8 months after the Care Standard was released. As there are no absolute requirements for sample size in qualitative research^16^ and the adequacy of a dataset is typically assessed based on its depth and richness rather than the size,^17^ we ceased data collection when we deemed to have enough information power (ie, the quality of the dialogue in the interviews was insightful, the themes were sufficient to address the study aim in a nuanced manner, GPs’ perspectives and experiences were diverse).^17^ We argue that using data saturation as a criterion for stopping data collection would be incongruent with our theoretical underpinnings and analytical approach because such a concept assumes that meaning is fixed in the data.^16^ The approach taken here, as many other approaches to qualitative research, assumes that meaning requires interpretation by subjective and situated researchers.^18 19^ All interviews were recorded, securely stored and transcribed using an online transcription software. NC deidentified transcripts to ensure confidentiality and checked them for accuracy.

### Analysis

Reflexive thematic analysis (RTA) was undertaken to explore meaning across transcripts,^20^ with relevance for exploring GPs’ perspectives of how the Care Standard could be implemented in clinical practice. RTA acknowledges researchers’ subjectivity as a resource rather than a problem, meaning that while the analysis is grounded in the data, codes and themes were produced by researchers through their engagement with the data.^20^ Consistent with RTA and the critical realist approach, the results presented here are subjective and influenced by our collective positionalities. Our background played a role in how we framed the questions, coded the data and interpreted the meaning of GPs’ responses. When interpreting the data, we considered experiential (eg, feelings), inferential (eg, aspects of the social world that can be inferred) and dispositional aspects (eg, factors that must exist for the quality statements to be followed through).^21^

While the phases of RTA are flexible and there are many ways of doing RTA, our analysis used the following approach: first, NC familiarised herself with the data by reading the transcripts. Here, she took notes of key points of interest, identified overlaps with her previous research^22^ and remained mindful of her subjectivity in her reading. Second, drawing from initial notes and interpretations acquired through the familiarisation phase, NC coded all transcripts using NVivo to organise the data. While the initial coding was primarily deductive and shaped by the TDF, the flexibility of RTA meant the analysis could be deductively informed by the domains of the TDF and inductively develop both semantic and latent codes, hence offering both descriptive and interpretative accounts of the data. As microdifferences in the dataset started to be captured, the coding became more inductive and produced more than 200 codes, which were subsequently grouped into broader categories. Third, NC used the categories to generate initial themes and reviewed them with SF and C-WCL. When describing each of the initial themes, NC used a taxonomy of implementation strategies to classify the strategies suggested by GPs.^23 24^ Finally, theme candidates were reviewed and refined by the whole research team during the writing of the final report. In this phase, the focus was on reflexive collaboration rather than consensus.^20^

## Results

We interviewed 16 GPs, with years of experience ranging from 12 to 35 years (mean–25 years) (see table 1). Only one participant had read the Care Standard prior to the interview and a further three had heard of it. We report three themes below, anonymising participants using numbers and illustrating the themes with additional quotes in tables2^4^. Barriers and strategies to overcome these, identified throughout the results, are summarised in table 5.

**Table 1:** GP-participants’ characteristics

	N	%
Age (years)		
35–45	2	12.5
46–55	6	37.5
56–65	7	43.75
Over 65	1	6.25
Sex		
Male	5	31
Female	11	69
State		
New South Wales	7	43
Western Australia	4	25
Victoria	2	13
Queensland	2	13
South Australia	1	6
Workload		
Full time	11	69
Part time	5	31
Practices in a regional or remote area		
No	14	87
Yes	2	13
Had heard about the care standard prior to the interview		
Yes	4	27
No	11	73

	Average (SD)	Range
Number of years in occupation	26 (6.8) years	12–35 years

GP, general practitioner.

**Table 2:** Themes, codes and quotes—theme 1: ‘I have my own little guidelines and standards and ways of doing things’—guidelines and standards are not relevant for experienced GPs

Codes	Quotes
No perceived need to consult or follow guidelines	No [I do not use guidelines or standards], I must say no. It’s personal. I sort of approach it from what I've seen and done in the past. G15 Interviewer: Are there any specific guideline that you follow? If so, how do you use them? GP7: No, I don’t. There are lots of guidelines. What I am is confident in my own practice. I know what to look for in a patient and recognise the signs, do the correct examination and do the appropriate investigations. GP13 Guidelines…are guidelines. They’re meant to be followed where possible. So you don’t always be prescriptive. One, people won’t do it. But two, you might miss things occasionally. GP5 I don't know specific ones, but I know…well, we’ve got something called the NPS here, which is, I think it’s the national pharmaceutical service. I think I know they put out some I don't know what are the important guidelines, but they put out suggestions. GP8 Well, I don't really [use guidelines]. So that’s interesting. I mean, most things don’t really have clinical guidelines. It’s just things I’ve learned over the years. But I suppose you know when you first start out, when doctors first start out, you know they are not confident with a lot of things, but as you get more experienced, you become more confident that things are going to get better, I suppose. GP11
Relying on experience gained over time	Interviewer: Do you use any specific clinical practice guidelines to help you to work with these patients? If so, how do you use them? GP3: No, just my own that I’ve accumulated along the years. I guess it’s just been experience and what works? (…) I guess as you have more experience, and you’ve been in it longer, you know what’s more common, and you can, you know, manage to do what you need to do in 15, 20 minutes, because that’s what you have to do in general practice. You can’t be taking an hour to see one patient most you know for most of the day. So you learn to do, you learn the shortcuts to do it. But if you were a new a GP, like, I remember when I was just starting out and back pain was like, What do I ask and how do I do this? I think what you’ve…what this layer that you're trying to implement, which I think would be very useful in health pathways, I think that’d be great for a junior doctor, or someone who’s just fresh out of medical school, done a couple of years and in the hospital system and going to general practice. I think that would have been very, very helpful to me. GP4 No, but one thing that does surprise me is the recommendation that, like things like pregabalin is not, is sort of not recommended in low back pain, because, you know, someone’s got sciatica, pregabalin is, in my experience, is the best drug to give them. GP8 It always takes a while of doing something for you to sort of get your own experience about what’s acute and serious and what’s not. (…) I think in making my assessment of whether it’s a serious problem or a mechanical problem, I feel quite confident in my ability to do that. And I think I just have my own little standard and my own sort of measured approach about the steps in which I like to do things. (…) I can always look them up if I need but I think I’ve just been doing this for so long, I sort of just have my own little guidelines and ways doing things. GP10 I think it’s, it’s hard to deny a trial of muscle relaxants. I think, you know, there’s some benefits, I think, in giving some people some valium at night to, you know, just sort of relax their muscles and give them a bit of taking some of the anxiety away. And while you’re also using the anti-inflammatory. So, you know, I found that to be very useful over the years for many people with back pain. (…) I think something that can never be overlooked in general practice is the placebo benefit of medications. That’s a real thing. You know, that people are taking a drug that’s been shown in studies not to be of much benefit, but for that patient, they may believe and it, you know, in fact, probably does help them, you know. So it’s one of those things that, in general practice, you end up becoming a bit non scientific a lot of the time, and relying on the patient’s perception of things rather than what some scientific study has shown. GP11 You know when to pull back and when to continue. And as a GP, that’s just experience over time. You have a patient who makes it quite clear that it will address something. You can only push it so far. Sometimes you’re just planting the seeds there and you’re meeting it first time, when they come back, you say, ‘Did just what do you think of what I said? Do you think it plays a part?’ GP12

**Table 3:** Themes, codes and quotes—theme 2: I agree with the standards but the system, clinicians and patients hinder my ability to enact them

Codes	Quotes
Healthcare system–competitive market	If you don’t give a patient what they want, it doesn’t mean that they’ll change their behaviour. They’ll change their doctor. So that's how our system operates. (…) You can only argue with people so long. And in our system, well, if you argue with somebody, you'll just never see them again. And the pharmaceutical industry is always pushing their own medications, so they’re pretty biased. GP11 They’re very good at marketing. There’s a reason that patients are willing to pay to see an osteopathic chiropractor cracks their back three times a week. They don’t want to go and see the GP once or twice. Perhaps we’re a bit more honest. (…) We’re not good at marketing as GPs. GP12 I guess that [quality statement about self-management and physical activity] may make the patient very disillusioned with service and with the health care with the medical profession, and that may drive them to other people, like allied health or physios or chiropractors or osteopaths or naturopaths. GP8
Healthcare system–remuneration for time	So there’s practical limitations on using documents the middle of a consult where the person is really there for one thing at a time and I have 15 minutes. Most of my colleagues might have five or 10 or less (…) Because it’s way the system remunerates, but if you're seeing a surgeon, you got 45 minutes, and the person is paying, so they’re valuing the time. (…) There’s a lot of other barriers in government. There’s politics involved. GP5 Score [of psychosocial screening tools], certainly will help us to know the situation a bit more, but I certainly can see the potential problem. It is time. I think time is just most, most important. I think it is certainly all good in theory, but I’m not sure that the time that we have will be able to let us explore all that in a 15-minute consult. GP7 You need a lot of time to talk to them about all these options. And sometimes, I think some GPs find it easier just to give them a prescription and a referral, because that’s really quick. (…) And then that has something to do with the Medicare rebate. You know, if the rebates were higher, people might be prepared to spend more time with patients because the remuneration is higher. GP8
Healthcare system–accessibility and affordability to services	The challenge I guess is if you use timeframes, like refer to a chronic multi-d chronic pain management programme. If they haven’t recovered by 12 weeks, then the timeframe to see a chronic pain unit would be like another 12 months from that point. So really, we would next be looking at like a physio-led private chronic pain programme. Yeah, but then it doesn’t exist, I guess otherwise. GP2 I think just there are some restrictions of accessing these treatments because of cost and wait time. So accessing, you know, physiotherapists and exercise physiologists and psychologists, for pain management psychologists, there is always a problem with the extra cost of the patient. GP6 Being rural and with less access to sort of other healthcare providers, I think would be a barrier. GP10
Patients’ context	If someone is old, and especially, you know, history of [physical] trauma, then I'll be more cautious and perhaps more willing to do imaging there**.** GP1 There is a lot of distress. And there’s the financial sort of fears as well, at the moment, in terms of the cost of everything. And if you can’t work and you lose your rental, and you know, there's a whole lot of other stuff that we don’t have control over. GP3 With our clinical space, we work with a pretty healthy, health-literate crowd. So it’s a bit easier with those, those simple barriers being a bit more easy. I’ve seen places where you have mixed sort of access to care, or language barriers, or health and equity barriers, money barriers, all these things impact on the ability to get going. GP5
Patients’ expectations	Patients’ strong expectations…our patients are being managed with strong opioids or analgesia, and they’re not really interested in what you're saying. They just want the script of analgesia. It becomes a bit hard because they already have made up their mind that this is the treatment. GP6 I’m prepared to discuss, but then the patients may not agree, may not understand, patients want to have a scan done. So, yes, I’m prepared to discuss, but it may not turn out that way. (…) Rather than arguing with the patients, wasting time at the end of the day, you still order the X-ray form. GP7 Now people are more aware, also with Dr Google and everything else before they come, oh, they walk in saying hello and I'd like an MRI. (…) That’s what I’ll get to so they listen more to Dr Google and their peers. GP13
Other healthcare professionals	I think the physios are very supportive, with a similar model. The chiropractors, I guess we don’t necessarily come from the same model of thinking. So that can hinder. I think other you know, managers, nurses, I think there’s I think generally people are on the same page. GP2 It’s not as simple as the doctor knowing the best medications. The crucial thing is, at the beginning, not even getting doctors not to put people on things that are going to get them addicted. GP11 I do in your full neuro examination. But most, a lot of doctors don’t do the neuro examination, and they end up saying, Oh, look, I’ll just send you for an MRI or whatever, which costs the government a lot of money. (…) Some doctors do that. GP13

**Table 4:** Themes, codes and quotes—theme 3: change the system and educate us better

Codes	Quotes
Need to change the system–improve reimbursement for GPs	So the incentive is going to be the Medicare rebate. If the Medicare rebate was higher, then doctors wouldn’t have to or want to see the number of patients, or, they would be said to see less patients an hour so they could spend more time with the patients to explain things like this. GP8 Rehaul, rehaul Medicare. Make Medicare a liable system. Patients are not willing to pay to see a doctor and pay equivalent to seeing a specialist or any other plumber, electrician or things like that. Where I’m at the bottom of the runway, we’re about this level of working in a sandwich shop. GP12 There is nothing you can do that’s going to make that happen, because unless the Medicare rebates go up for the amount of time you spend with the patient or what you do significantly. GP13
Need to change the system–use incentives and disincentives	So you probably have to give GP some sort of incentive to deprescribe people, because you can only argue with people so long. In the long run, is some incentives for the patients (…) doctors or the government could say, there’s now an opportunity for people, if they come off their medications, or reduce their medications to have some physiotherapy and pilates and things like that. GP11 Financial and, give doctors, you’ll get them attending. If there’s a financial incentive, they’ll find time. GP12 I think CPD [continuing professional development] points is always you have to do them. (…) you can offer a financial incentive to look at it, that might work. Maybe through their, through their medical legal, through their indemnity, medical insurance, webinars and meetings that we can go to via MDA [Medical Directory of Australia]. GP4
Need to change the system–more publicly funded allied health services	With the care plan, there could be more Medicare-funded visits [for allied health] in a calendar year. GP1 I think by making more services available, making it easy to access clinics in the public system and the private. Getting Medicare to supplement or to cover visits to psychology, or physiotherapist. GP6 If we think about how much money the economy is losing because of this problem, I think surely there must be a place for some free hydrotherapy exercise for a period of time. I do feel that if patients has access to something that was completely free, not just subsidised, but completely free for a certain amount of time. GP9
Educate GPs better	Evidence that this actually works, having access to studies that show what happens when you do prescribe antidepressants or anticonvulsants or benzos or opioids and vs simple analgesics in terms of what percentage of patients get better with or without those things. GP8 Overhaul Medicare and give us, give us more time in training, perhaps train us better. We do a lot of education, continued education as GPs, but this in particular would be really good to learn more about [psychosocial assessment]. (…) I think in a lot of conditions they’re addressing that and making people realise it is an important contributing factor. GP12 Starting with medical students, listening and communication. It was 99% biomedical in my day. GP14
Developing practical tools and interactive assistance	If there could be a template or something with the assessment tool, and that can be, put into our best practice software. If there was some shortcuts that way, so that we could do a more detailed physical examination and spend less time on writing everything down, and the questions to ask as well. GP3 RACGP has algorithms of guidelines as to how you should manage, for example, breast lumps. Maybe it could be incorporated in association with the RACGP to formalise this. GP4 Find the best way to remind you to do things in general practice…auto checks so I can, I can type in low back pain, it will come up with all the guideline, well, not the, but the points to discuss. GP8
Educating patients	I guess also something that's written information that you can give to a patient to go and have a read about this, and then we’ll come back and discuss it that would be helpful. GP2 We should encourage type thing as a handout (…) just having something practical about it, making it available, make it free, making it a patient, a friendly component to it. GP5 I guess patient information sheets. Being able to show the patient, what statistics, what percentage of low back pain is serious, what percentage you’ll see something on there, something meaningful on investigations, what percentage get better with conservative treatment. GP8
Dissemination of LBP care standards	To make us aware, aware of the guidelines, through meetings. GP7 RACGP, NPS [National Prescribing Service], national prescriber. It’s something that the PHNs [Primary Health Networks] could circulate. (…) It could be in the media, the Australian Doctor media and medical Republic. (…) Some of this messaging could be circulated in workplaces with high burden of low back pain. GP2 I think better promotion of them because I have seen them before, but I can’t say, to be honest, that they’re first and foremost in my mind. I think sort of making people more aware that they exist would be helpful. GP10
Audit	If GPs had to do an audit of the people with back pain. (…) to monitor your own assessments of things and look back and reflect on how well you did things or how poorly you did that. GP3 I think peer pressure, so you’re being educated with your colleagues, perhaps in the with their own practice, or their own division of general practice (…) then people could have a look at what all different people are prescribing, and seeing the range of things that are prescribed. GP11 A lot of the GPs won’t do it because the watchdog, the PSR [Professional Services Review]. You have too many long consultations, more long consultations than your fellow GPS. Oh, so they audit you so they won’t do it. GP13

GP, general practitioner; LBP, low back pain.

**Table 5:** Summary of barriers and strategies identified and categorised according to each of the TDF domains

Barriers (TDF domain)	Potential implementation strategies for addressing barriers
Perceived unnecessariness of reading or using the care standards because of the experience and skills gained over time (knowledge, skills)	Identify a ‘shared vision’ for why engagement with the care standards is needed, use opinion leaders
The perception that standards and guidelines are beneficial for junior colleagues only (knowledge, skills)	
Variation in practices and messages between clinicians (social influences)	Involve as many clinicians as possible in implementation efforts, from all clinical backgrounds, in order to promote a cohesive approach to LBP care Develop stakeholder relationships to increase the reach of the dissemination of the care standards
Potential lack of critical self-reflection in current practice (beliefs about capabilities, goals)	Audit and feedback, self-reflection as part of education strategies
Fear of not meeting patients’ expectations and/or losing them (beliefs about consequences)	Educating the community on what care patients with LBP should expect when seeking care (eg, through mass media campaigns, patient-friendly handouts and education resources)
Knowing about the standards is not enough to prompt the intention to meet standards more often (Intentions)	Supporting clinicians through interactive assistance (e.g., reminders, embedding assessment tools in existing systems/software, developing practical tools)
Low optimism about how the standards will benefit patients due to systemic challenges (Optimism)	Advocacy for systemic changes, providing education on how the standards can benefit patients
Patients’ circumstances (e.g., health literacy, language barriers, age, ability to afford LBP care) and expectations (Memory, attention and decision processes, Environment)	Education strategies that prompt dispositional knowledge about how to attune the standards to patients’ circumstances, education on how to navigate patients’ expectations
Lack of time and insufficient remuneration for time (Reinforcement)	Advocacy for systemic changes
Access to publicly funded services (e.g., multidisciplinary services, allied health, exercise programs, particularly difficult in rural/remote areas) (Behavioural regulation)	Advocacy for systemic changes
Dealing with all these barriers when providing care can be frustrating (Emotions)	Advocacy for systemic changes, developing stakeholder relationships (e.g., peer support)

LBP, low back pain; TDF, Theoretical Domains Framework.

### Theme 1: ‘I have my own guidelines and ways of doing things’—guidelines and standards are not relevant for experienced GPs

I need to have enough kind of intrinsic understanding of something just to do it, and then look at the guidelines where I’m uncertain. GP2

Most GPs reported not following any specific guidelines or standards when working with patients with LBP. The experience and skills gained over time—what works, how patients respond to medications and treatment approaches, what questions to ask, what shortcuts to take—seemed to remove the need for, and prompt certainty about, the unnecessariness of seeking advice from the latest evidence on how to provide care. Overall, participants seemed to believe they met the care standards most of the time and some highlighted that the Care Standard would be relevant to medical students and junior doctors rather than experienced GPs. While the certainty and confidence GPs displayed during the interviews regarding their clinical skills can be interpreted as helpful (eg, it may stem from reasoned clinical judgements, it can engender trust in patients), it might also be problematic within the broader context of implementing the Care Standard; it suggests that engagement with the quality statements and potential consequent improvements in practice is unlikely. The observation that only one GP had read the Care Standard at the time of the interview supports this interpretation; most GPs were not aware of it, and those who had heard of it did not seem compelled to read it.

### Theme 2: I agree with the standards but the system, clinicians and patients hinder my ability to enact them

Time, remuneration for the time spent. Conflict with messages from other practitioners. Patient factors that might bring like their previous experiences or experience with friends or family. Their understanding of results of previous imaging and things that have worried them previously. There’s lots of things. GP2

Although all GPs agreed with the quality statements of the Care Standard and mostly believed they could lead to improvements for patients and the healthcare system if consistently implemented in primary care, they discussed how the healthcare system, patients and other healthcare professionals impacted their ability to meet the standards. At a macrolevel, remuneration for time and accessibility to timely and publicly funded services were often reported as the main factors influencing the decision-making surrounding the quality statements. Discussions about such factors incited deliberations about their professional practices, through which they position themselves (and at times, other healthcare professionals) as victims of neoliberalist strategies such as privatisation, competitive markets and reduced public expenditure on health services:

They don’t, because their other pressures are higher, meet your quotas, earn the money. See a certain number of patients. Don’t have 85 people in the waiting room waiting to see you. Practising good medicine takes time, and private equity doesn’t want you to spend the time. They want you to make the money. And that’s the problem, very difficult. GP13

I guess that [quality statement about self-management and physical activity] may make the patient very disillusioned with service and with the health care with the medical profession, and that may drive them to other people, like allied health or physios or chiropractors or osteopaths or naturopaths. GP8

There’s a pain clinic which also has allied health, psychologists, physiotherapists attached to it, and it gives a very good multidisciplinary approach to people with chronic pain. So it’s a great service. It’s just very, very hard to access. (…) I think it’s under resourced and it’s too busy. GP10

 GPs also highlighted how factors related to patients’ context, such as their age, levels of anxiety and distress, financial circumstances, language, health literacy and rapport, influenced their willingness to meet the Care Standards. For instance, some reported going against them and referring patients to imaging when it is not clinically necessary in order to reduce anxiety, and others discussed avoiding referring patients to physical and psychological treatments if patients cannot afford them or waiting times are long. Similarly, patients’ expectations regarding what care they should receive were often reported as important hindrances to implementing the Care Standard, with some GPs fearing losing their patients. GP14 reflected on a recent appointment with a patient, who refused to pay for a consultation that involved education and advice for physical therapies:

She didn’t want to pay the bill because I said her interpretation was, ‘but [GP14] couldn’t help me’. There’s that sort of thing. That’s another barrier that’s worth putting in there somewhere, is giving people a piece of paper that’s a prescription or a referral, looks like you had done something. People do sometimes come out of these consults with the impression that was a waste of time. ‘The doctor didn’t do anything for me’ when they may have spent 25 minutes doing these beautiful education guidelines.

In addition to commenting regularly on their own practice, GPs discussed the practices of other healthcare professionals and, at times, discussed them as hindrance factors:

I’ve had too many disagreements with other professionals. I think some have come to the wrong diagnosis of what the cause of the back pain is. And possibly some people have explained it badly to the patient, and so you have to re-educate them on it. And maybe they’ve suggested mobilisation or injections or back surgery when the patient actually doesn't need to have this. GP3

Overall, our analysis of GPs’ responses suggests that enacting the Care Standard overlaps with various political, situational, material, social and cultural factors embedded (although at times, hidden) in their practices and intertwined with the healthcare system, patients and other healthcare professionals. Notably, change in relation to these might be obscured through the evocation of GPs’ ‘own guidelines and ways of doing things’.

### Theme 3: change the system and train us better

Many GPs expressed low optimism about the standards improving patient outcomes due to systemic challenges. In alignment with theme 2, many of the implementation strategies GPs suggested were related to funding-related healthcare system changes, for example, improving GPs’ reimbursement for time spent, investing in publicly funded allied health services and using (dis)incentives to prompt doctors to meet the standards or attend to training sessions on the Care Standard:

Rehaul, rehaul Medicare. Make Medicare a liable system. Patients are not willing to pay to see a doctor and pay equivalent to seeing a specialist or any other plumber, electrician or things like that. I’m at the bottom of the runway, we’re about this level of working in a sandwich shop. GP12

Making more services available, making it easier to access clinics in the public system, as well as the private. Getting Medicare to supplement or cover visits to psychology, or physiotherapist for a certain amount of time. GP6

You probably have to give GP some sort of incentive to deprescribe people, because you can only argue with people so long. (…) In the long run, some incentives for the patients, so that, if they didn’t, if the doctors or the government could say, ‘there’s now an opportunity for people, if they come off their medications, or reduce their medications to have some physiotherapy and Pilates and things like that’. (…) In England, when they did it, they had to pay the GPs to go to a workshop with academic detailers [academics who are not representing pharmaceutical companies and visit GPs to inform them about the latest evidence of medications]. GP11

Elaborating on these suggestions often seemed to evoke a sense of frustration, tiredness and lack of hope*—“I can’t see that happening”* GP7. Contrastingly, GPs seemed to be more positive about potential implementation strategies related to (preferably hands-on) training and education:

Sometimes using SMART goals is good, sounds good on paper, but then how does it work in the real world? What motivational barriers or factors do they have in their life? So maybe some strategies to address that if there are any major barriers. GP5

Other suggested education-related strategies included embedding the Care Standard into continuous professional development courses, improving communication training in undergraduate courses, role-playing, discussions with peers and having specialists and academics visit clinics to provide training. Developing stakeholder relationships (eg, disseminating the Care Standard through peak bodies, Health Pathways, partnering with pharmaceutical companies to sponsor educational meetings), supporting clinicians through interactive assistance (eg, reminders, embedding assessment tools in existing systems/software), engaging/educating patients (eg, developing handouts) and using evaluative strategies such as auditing were also suggested as potential avenues for implementing the Care Standard in primary care.

## Discussion

Our study identified factors that may impact GPs’ ability and willingness to meet the Care Standards. Overall, GPs did not consider it to be relevant to them. Notably, some of the structural strategies suggested by GPs, such as changes in general practice reimbursement and increasing public funding for allied health services, will require a shift from thinking about spending on health services as a burdensome cost to perceiving it as an investment that produces social benefits and promotes economic growth. While previous research supports the latter view,^25^ major system and policy changes will require political commitment, advocacy and leadership. Nonetheless, our results also highlight other potential strategies that could be more easily implemented: offering a range of tailored educational opportunities to GPs, developing stakeholder relationships to assist dissemination, supporting clinicians through interactive assistance, engaging/educating patients and using evaluative strategies such as auditing. Yet, these strategies might be more likely to be successful if GPs are able to see how the standards are relevant to them and how they can be used in practice.

The barriers illustrated in themes 1 and 2 broadly align with those reported in a previous review on barriers to primary care clinicians’ adherence to guidelines for the management of LBP.^26^ That review highlighted clinicians’ perceptions that their practical experience superseded guidelines and identified time constraints as a barrier to implementing guidelines.^26^ Likewise, a review of factors influencing the implementation of evidence-based guidelines for osteoarthritis in primary care has shown that the argument of ‘best practice’ is not enough to achieve buy-in to implementation, indicating that the need for moving beyond such an argument is not limited to LBP.^27^ Interventions and recommendations that challenge professional identity, contradict habitual practice and require skilful communication might be particularly hard to implement not only for GPs, but also other primary care professionals such as physiotherapists.^28^

GPs suggested several strategies that could be used alongside education when implementing the care standards. This is consistent with the results of a review of implementation initiatives targeting LBP in Australia, which indicated that the strategy of training and educating clinicians seems to lead to better outcomes when used alongside other strategies rather than used alone.^29^ That same review identified that studies that aimed to increase the adoption of more than one behaviour related to LBP management improved the adoption of at least one behaviour, but not all. In light of such findings, considering that the Care Standard has eight Quality Statements and that GPs are time-poor, it might be useful (and potentially more feasible) to create multimodal educational and training opportunities that enable GPs to choose the quality statement they think they need more help with and/or that might be particularly problematic to enact in their context. Some GPs indicated that some of the quality statements were more challenging than others (eg, listening to patients’ concerns as part of the psychological assessment, supporting patients’ goals as part of encouraging self-management, knowing what medications to prescribe based on the latest evidence), with relevance for adapting training opportunities to their needs. Notably, adapting and tailoring innovations to meet local needs of health professionals can be effective.^30^ The perceived unnecessariness of consulting guidelines and standards presented in our results further supports the importance of a GP-tailored approach to training and education, preferably alongside other strategies that enable GPs to see how the Care Standard is relevant to them, such as using opinion leaders.^31^ Some participants suggested education strategies could be further supported by self-reflection, academic detailing,^32 33^ financial incentives to attend continuing education opportunities and the offering of both online and face-to-face delivery modes.

GPs’ argument for an integrated set of strategies aligns with evidence that multifaceted strategies appear to have a greater impact than single-component interventions when implementing guidelines and standards.^34^,^36^ It would, therefore, be equally important to: (1) engage with peak bodies early, strengthening relationships to boost dissemination of the Care Standard; (2) promote its quality statements across consumer communities and the general population through consumer organisations and mass media campaigns; (3) audit current practice and provide feedback to GPs (previously done to improve LBP care in Australia^37^,^40^) and (4) develop tools to embed in current systems and software to prompt clinicians to enact quality statements. The latter will require input from GPs as part of its development to pre-emptively address barriers identified in previous tools that were developed to assist GPs with LBP management. For instance, GPs have found a Clinical Decision Support System tool designed to assist LBP management condescending and not sufficiently dynamic.^41^ Likewise, including consumers in the development of mass media materials might be equally important, considering that messages that align with the latest evidence can have unintended consequences and be met with resistance.^42^

### Limitations

There are important limitations to deliberate when considering the transferability of these results to different contexts. First, we interviewed GPs who work in Australia and, therefore, the results presented here may not be applicable elsewhere because healthcare systems and care pathways for LBP vary across countries. Yet the Care Standards were developed based on the latest worldwide evidence for LBP and it is possible that GPs who work in healthcare systems similar to the Australian system may share similar views on how guideline concordant care for LBP can be implemented. Second, although we made efforts to include GPs with different levels of experience, the results presented here may not represent those who have recently entered general practice. Third, we only interviewed GPs and therefore the results presented here may not reflect the experiences of other healthcare professionals, with relevance for exploring their experiences in future research. Fourth, we did not advertise our study in public healthcare institutions because most GPs in Australia (90%) work in private settings,^43^ with relevance for the results presented here. Additionally, the recruitment of GPs in public settings would require us to seek ethical clearance from each of them. We chose to recruit via social media and a recruitment service due to efficiency and available funds. Fifth, only one of the GPs had read the Care Standard before the interview. Our results could have been different if most GPs had been aware of the full content of the Care Standards prior to the interviews. Future studies could be conducted with GPs who are familiar with the recommendations to explore their views on how the Care Standard could be implemented. Although this is a potential limitation, the GP who had read it reported facing similar challenges to those who did not. Lastly, while the authors’ collective positionalities are not a problem to be managed or gotten rid of according to RTA principles,^20^ it is important to acknowledge that most of the research team has not worked clinically with people with LBP in recent years.

## Conclusions

The multidimensional nature of the challenges GPs face, and the strategies they suggest, calls for a multimodal approach to implementing the Care Standards, including system changes.

## Supplementary material

10.1136/bmjph-2025-002564 online supplemental file 1

10.1136/bmjph-2025-002564 online supplemental file 2

## Data Availability

No data are available.
