# Supplementary material for: ‘Overhaul Medicare and perhaps train us better’: a qualitative study of primary care general practitioners’ perspectives on how to implement the low back pain clinical care standards
Source: BMJ Public Health. 2025 Sep 8;3(2):e002564. doi: 10.1136/bmjph-2025-002564 (PMC12421184; doi:10.1136/bmjph-2025-002564)
Supplement: online supplemental file 1 [file bmjph-3-2-s001.pdf]

Figure 1. Summary of the eight quality statements of the Low Back Pain Clinical Care Standard

Quality statement 1 – Initial clinical assessment
The assessment of a patient with a new presentation of low back pain symptoms, with or without leg pain or other neurological symptoms, focuses on screening for specific and/or serious pathology and consideration of psychosocial factors. It includes a targeted history and physical examination, with a focused neurological examination when appropriate. Arrangements are made for follow-up based on an evidence-based low back pain pathway.

Quality statement 2 – Psychosocial assessment
Early in each new presentation, a patient with low back pain, with or without leg pain or other neurological symptoms, is screened and assessed for psychosocial factors that may affect their recovery. This includes assessing their understanding of, and concerns about, diagnosis and pain, and the impact of pain on their life. The assessment is repeated at subsequent visits to measure progress.

Quality statement 3 – Reserve imaging for suspected serious pathology
Expectations of imaging and its limited role in diagnosing low back pain are discussed with a patient. Early and appropriate referral for imaging occurs when there are signs or symptoms of specific and/or serious pathology. The likelihood and significance of incidental findings are reported and discussed with the patient.

Quality statement 4 – Patient education and advice
A patient with low back pain is provided with information about their condition and receives targeted advice to increase their understanding, and address their concerns and expectations. The potential benefits, risks and costs of medicines and other treatment options are discussed, and the patient is supported to ask questions and share in decisions about their care.

Quality statement 5 – Encourage self-management and physical activity
A patient with low back pain is encouraged to stay active and continue, or return to, usual activity, including work, as soon as possible or feasible. Self-management strategies are discussed. The patient and clinician develop a plan together that includes practical advice to maximise function, and limit the impact of pain and other symptoms on daily life. The plan addresses individual needs and preferences.

Quality statement 6 – Physical and/or psychological interventions
A patient with low back pain is offered physical and/or psychological interventions based on their clinical and psychosocial assessment findings. Therapy is targeted at overcoming identified barriers to recovery.

Quality statement 7 – Judicious use of pain medicines
A patient is advised that the goal of pain medicines is to enable physical activity, not to eliminate pain. If a medicine is prescribed, it is in accordance with the current Therapeutic Guidelines, with ongoing review of benefit and clear stopping goals.

Anticonvulsants, benzodiazepines and antidepressants are avoided, because their risks often outweigh potential benefits, and there is evidence of limited effectiveness. Opioid analgesics are considered only in carefully selected patients, at the lowest dose for the shortest duration possible.

Quality statement 8 – Review and referral
A patient with persisting or worsening symptoms, signs or function is reassessed at an early stage to determine the barriers to improvement. Referral for a multidisciplinary approach is considered. Specialist medical or surgical review is indicated for severe or progressive back or leg pain that is unresponsive to other therapy, progressive neurological deficits, or other signs of specific and/or serious pathology.
