# Supplementary material for: ‘Overhaul Medicare and perhaps train us better’: a qualitative study of primary care general practitioners’ perspectives on how to implement the low back pain clinical care standards
Source: BMJ Public Health. 2025 Sep 8;3(2):e002564. doi: 10.1136/bmjph-2025-002564 (PMC12421184; doi:10.1136/bmjph-2025-002564)
Supplement: online supplemental file 2 [file bmjph-3-2-s002.docx]

# **Appendix 1 – Interview guide**

**Semi-structured interview guide**

*Preamble: Thanks for agreeing to participate in our study. As I mentioned earlier, we are interested in understanding your views on how the low back pain care clinical standard could be implemented in practice/primary care. There are no right or wrong answers or things to say. We are keen to hear your thoughts and opinions, either positive, negative or neutral. During the interview I may ask for clarification using questions such as “take me through your experience” or “can you please further elaborate on this”. Please stop me at any time if you need to take a break or if you want to end the interview for any reason. Also, please remember that you don’t have to answer every question – just let me know if you want to move on from something.*

1. **I would like to start by asking you to tell me how you manage patients who present with low back pain. What is your usual routine once the patient is in the room?**

*Follow up questions:*

- 1. *What skills do you think a general practitioner (GP) needs to work with these patients?*
  2. *What do you think are the challenges when working with these patients?*
  3. *What do you think you do well when working with these patients?*
  4. *Thinking more broadly (e.g., context, resources), what factors or resources could help you to work better with these patients?*
  5. *Do you use any specific clinical practice guideline/s to help you to work with these patients? If so, how do you use it/them?*
  6. *Have you used the low back pain clinical care standard before?*

1. **For the rest of the interview, I will ask you specific questions about the low back pain clinical care standard. I will give you some time to look at the quality statement. [Interviewer randomly selects one recommendation].**

**What comes to mind as you read this?**

*Follow up questions:*

*2.1. What stands out to you?*

*2.2. Is there anything you find contentious or challenging?*

**Knowledge**

1. **What do you know about this quality statement already?**

*Follow up questions:*

*3.1. Are there any gaps in what you know about it? If so, what are they?*

*3.2. What things would be most helpful to you to learn more about this quality statement?*

**Skill**

1. **Do you know how to ___(re-state the quality statement)____?**

*Follow up question: How prepared do you feel regarding how to do X?*

*(If the participant feels unprepared – What do you think that would help you to develop/strengthen these skills? e.g., mentoring, training, materials, pathways, time)*

**Social/Professional role**

1. **To what extent do you think following this quality statement is part of your professional role?**

*Follow up questions:*

*5.1. Are there any people or institutions who have a role in supporting/ensuring this quality statement is followed?*

*5.2. (if they don’t think following the quality statement is their role) – Is there anything that would change your mind about this?*

**Beliefs about capabilities**

1. **How confident do you feel about following this quality statement?**

*Follow up questions:*

*6.1.*

- 1. *If confident – how confident are you in maintaining or enhancing your existing practice?*
  2. *If not confident – thinking broadly, what things would help you to feel more confident? (e.g., not only micro, but meso and macro aspects – organisation, health system)*

*6.2. How difficult/easy is it to follow this quality statement?*

**Beliefs about consequences**

1. **What do you think would happen if you were to always follow this quality statement?**

*Follow up questions:*

*7.1. How are your patients likely to react?*

*7.2. What impact do think this would have on your practice/workplace/colleagues?*

*7.3. What impact do you think this would have on the healthcare system more broadly?*

If the participant raises positive consequences, ask about negative consequences and vice-versa.

If/when they raise negative consequences, as what could help to manage the perceived negative consequence.

**Goals**

1. **How motivated do you feel about ___(re-state the quality statement)__?**

*Follow up question: Thinking broadly, what would need to happen to make following this quality statement a priority?*

*(If the person is already following the quality statement – Thinking broadly, what would need to happen for you to increase the extent to which you follow this recommendation)? What things could help to motivate other GPs?*

**Intentions**

1. **Now that you have seen this quality statement in the low back pain clinical care standard, how much do you intend to follow/follow more often the ___(re-state the quality statement)__?**

*Follow up question: Are there any strategies you intend to implement in your daily routine to remind you to ___(re-state quality statement)____ more often?*

**Optimism**

1. **How confident are you that doing following this recommendation will lead to best outcomes for patients?**

*Prompts:*

*10.1. Can you elaborate on how do you think this quality statement would improve/decrease the care people receive if followed by GPs?*

*10.2. What things could help GPs to feel more confident about this?*

**Memory, attention, decision processes**

1. **Are there any things that get in the way of you deciding to ___(re-state the quality statement)__?**

*Follow up questions:*

*Are there any situations or factors you think that would impact your decision to ___(re-state quality statement)___?*

*Prompts:*

*11.1. What is it about these situations/factors that make it difficult to ___(re-state the quality statement___?*

*12.1. What could help to overcome these barriers?*

**Reinforcement**

1. **Are there any incentives (or disincentives) for you to ___(re-state the quality statement)__?**

If disincentives are discussed – What would counter these, if anything?

**Behavioural regulation**

1. **Are there things you would need to ___(re-state the quality statement)__?**

*Follow up questions:*

*13.1. Are there ways of working or strategies that would help you to ___(re-state the quality statement___?*

*13.2. What do you think is needed to ensure that GPs ___(re-state the quality statement)__?*

**Social influences**

1. **I now want you to reflect about your peers, managers, professional groups, patients and any other relevant groups. To what extent do they encourage or hinder your ability to *___(re-state the quality statement)___?***

*Follow up questions:*

*14.1. How do ___(state name of the group)___ make your feel about to ___(re-state the quality statement___?*

14.2. How do you think these influences can be managed?

**Emotions**

1. **To what extent following this quality statement would be emotionally challenging or an emotionally charged situation?**

*Follow up question: How do you feel when you are discussing/explaining/doing ___(re-state the quality statement)__with your patients?*

**Environment**

1. **What organisational or healthcare system factors help or hinder you to *___(re-state the quality statement)___, if any?***

*Follow up question: How could your organisation or the healthcare system more broadly help you to follow ___(re-state the quality statement___?*

**Closing questions**

1. **Are there any types of patients for whom acting on this quality statement may be more difficult?**
2. **Is there anything else you would like to add that we haven’t discussed today?**
